# Leisure time physical activity and depressive symptoms among adolescents in Sweden

**DOI:** 10.1186/s12889-020-09022-8

**Published:** 2020-06-26

**Authors:** Li Ma, Curt Hagquist, Annette Løvheim Kleppang

**Affiliations:** 1 Centre for Research on Child and Adolescent Mental Health, Karlstad University, Universitetsgatan 2, 651 88 Karlstad, Sweden; 2 Department of Public Health and Sport Sciences, Inland Norway University of Applied Sciences, Campus Elverum, Terningen Arena, PO Box 400, 2418 Elverum, Norway

**Keywords:** Physical activity frequency, Depressive symptoms, Adolescents, Sweden

## Abstract

**Background:**

Mental health problems have increased noticeably among adolescents. Physical activity has been identified as an important factor in the promotion of mental health. The purpose of this study was to explore how leisure time physical activity was associated with depressive symptoms among adolescents in Sweden.

**Methods:**

Using binary logistic regression we analyzed Swedish data collected as part of the Children of Immigrants: Longitudinal Survey in Four European Countries. The complete sample used for analysis consisted of 3787 adolescents (including 1855 boys and 1932 girls).

**Results:**

Adolescents who participated in physical activity during their leisure time on a daily, weekly, or monthly basis had substantially lower odds of often feeling depressed than those who were physically inactive. This pattern applied to both boys and girls. Relative to boys, girls had significantly higher odds of often feeling depressed.

**Conclusions:**

The findings of this study suggested that participation in physical activity regularly during leisure time was associated with a lower level of depressive symptoms among adolescents in Sweden. Because of the cross-sectional study design conclusions about causality cannot be drawn. Future research based on longitudinal data is needed to further explore the mechanisms behind the association. This study calls for recognition of the value of physical activity in promoting mental health among adolescents.

## Background

Mental health problems, including symptoms of depression, have been identified as an important worldwide public health issue [1]. Mental health problems have increased among adolescents globally as well as in Sweden [2, 3]. A systematic review of the international literature reported that internalizing mental health problems have increased over time, especially among adolescent girls; however, the findings for boys are mixed [2].

Identifying factors that can reduce mental health problems is important. Physical activity (PA) has been characterized as an important and positive behaviour in the promotion of mental health [4–7]. Existing research has reported gender differences in both physical activity and depressive symptoms, with girls being less physically active while having higher levels of depression symptoms than boys [8, 9].

Empirical research on the association between participation in PA and mental health has provided evidence that physical activity is beneficial for mental health among adolescents [10, 11]. A recent meta-analysis showed that higher levels of PA was associated with lower odds of developing depression not just among adolescents, but also among adults and the elderly, regardless of age and gender [12].

Existing studies for the Nordic countries demonstrated inconsistent results. Studies in Finland reported a negative association between participation in PA and emotional, social and attention problems among both boys and girls [13]. Studies in Iceland also showed that PA reduced the risk of depressed mood among both boys and girls [14]. Studies in Denmark found that girls with low levels of PA during adolescence had an increased risk of poor mental health during young adulthood. However, this association did not hold for boys [15]. Studies on Norwegian adolescents reported a weak association between PA and mental health problems. Researchers found that only a high amount of PA (at least 11 h per week) was associated with a significantly lower risk of psychological distress [16]. In addition, adolescents who were physically active in a sports club showed a lower level of depressive symptoms compared to those who were inactive [17].

Studies in Sweden showed that participation in PA was closely associated with adolescents’ physical self-esteem and weight control [18, 19], whereas it had no clear association with their psychosomatic complaints [20]. So far, we have little specific knowledge about how participation in PA is related to adolescents’ depressive symptoms. The purpose of this study was to explore the association between physical activity frequency at leisure time and depressive symptoms among Swedish adolescents.

## Methods

### Sample

Data used for analysis came from the Swedish section of the Children of Immigrants: Longitudinal Survey in Four European Countries (CILS4EU) study, which includes data from Germany, the Netherlands, Sweden, and England [21, 22]. The data include not only children of foreign-born parents, but also children of native-born parents. That is, the survey is generalizable to the general population of youth in each respective country [21, 23].

During the period 2010–2011, 5025 students in grade eight (aged around 14–15 years) in Sweden responded to the CILS4EU questionnaire [22]. Of them, 4531 (90%) were followed up in 2012 when they were in grade nine [22]. Due to missing data, the final analytical sample size of this study was 3787 adolescents (including 1855 boys and 1932 girls). Among them, 30% had both parents foreign born. Given that the corresponding figure for the total population was 21% in 2012 [24], our sample was over-represented by children of immigrants.

### Measures

The outcome measure of this study was adolescents’ depressive symptom during grade nine (see Table 1). In the questionnaire, the students were asked how often the statement “I feel depressed” was true. There were four response categories, including “often”, “sometimes”, “rarely” and “never”.

**Table 1:** CILS4EU - Sweden waves 1–2: Questions, response alternatives and variable definitions

Questions	Response alternatives (scales)	Variable definitions	Waves
**Outcome variable**			
Depressive symptoms in grade nine			
How often are each of these statements true about you?			
I feel depressed.	Often true, sometimes true, rarely true, never true	Often (often true), non-often (sometimes true, rarely true, never true)	2
**Main explanatory variable**			
Physical activity (PA)			
How often do you do sports or go to the gym?	Every day, once or several times a week, once or several times a month, less often, never	Every day, once or several times a week, once or several times a month, physically inactive (less often or never)	2
**Control variables**			
Smoking			
How often do you smoke cigarettes?	Every day, once or several times a week, once or several times a month, less often, never	At least once a month, less often, never	2
Future education plan			
What are you planning to do after this school year (after the summer holidays)?	Upper secondary school, academic track; upper secondary school, vocational track; upper secondary school, provisional track; I will not study but intend to work instead; something else	Academic track, vocational track, something else	2
Parents' education			
Did your father/mother complete primary school? Did your father/mother complete secondary school? Did your father/mother complete university?	Yes, no, don't know	Both tertiary educated, one tertiary educated, neither tertiary educated	2
Family structure			
Do you live with both your biological parents in one home? Do you also live in another home on a regular basis? Who lives in this second home? How much of the time do you usually live in this second home?	Yes, no; Yes, no; Biological mother, biological father, …; More than half the time, about half the time, less than half the time, almost never	Two-parent household, one-parent household, equally shared custody, unequally shared custody	1
Gender			
Are you a boy or a girl?	Boy, girl	Boy, girl	2
Depressive symptoms in grade eight			
How often are each of these statements true about you?			
I feel depressed.	Often true, sometimes true, rarely true, never true	Often (often true), not often (sometimes true, rarely true, never true)	1

The students also reported how often they did sports or went to the gym during leisure time in grade nine. The response had five categories, including “every day”, “once or several times a week”, “once or several times a month”, “less often”, and “never”. In this study, we used the term physical activity at leisure time to represent the frequency that adolescents did sports or went to the gym. This variable was as the main independent variable of this study. It was categorized into four frequency levels: every day, once or several times a week, once or several times a month, and physically inactive (including those who do sports or go to the gym less often and those who never do sports or go to the gym).

A few variables were controlled for, including smoking, future education plan, parents’ education, family structure, gender, and depressive symptoms in grade eight. Smoking was categorized into three frequency levels based on adolescents’ smoking frequency: at least once a month, less often, and never. Adolescents’ future education plan was measured with their responses to the question “What are you planning to do after this school year?”. Their responses were grouped into three categories: academic track of upper secondary school, vocational track of upper secondary school, and something else (including provisional track, intending to work, or something else). Parents’ education was categorized as both tertiary educated, one tertiary educated, and neither tertiary educated. A family structure variable was constructed based on adolescents’ responses to whether they lived with both parents in one household. For those who did not, we tracked their responses to whether they lived in another home, with whom, and how much time they lived in this second home. Given that our data from wave two did not provide relevant information, we classified family structure based on the students’ responses in wave one. Those living with both parents were categorized as two-parent household, those living with only one parent were categorized into one-parent household, those who lived under equally shared custody of parents were categorized as equally-shared custody, and those who spent more time with one parent and less time with the other were categorized into unequally-shared custody. Additionally, we included adolescents’ depressive symptoms during grade eight into our analysis.

### Analytical strategy

We applied binary logistic regression analysis to estimate how adolescents’ participation in PA during leisure time was associated with frequency of depressive symptoms during grade nine. Adolescents who participated in PA every day were used as the reference category. The original four-category variable on depressive symptoms was dichotomized and we calculated adolescents’ odds of often feeling depressed versus other levels of depressive symptoms (i.e. sometimes, rarely and never feeling depressed). Smoking, future education plan, parents’ education, family structure, gender, and depressive symptoms in grade eight were controlled for due to their importance in predicting mental health problems. The estimated results were presented in the form of odds ratios.

Furthermore, to explore whether the association between leisure time PA and depressive symptoms vary across genders, we estimated the interactive effect of physical activity frequency and gender, while other variables were controlled for.

## Results

### Descriptive results

Table 2 presents the variables used for analysis in the logistic regression model. Among the 3787 adolescents who entered our observation, 262 often felt depressed, amounting to 7% of the total sample.

**Table 2:**
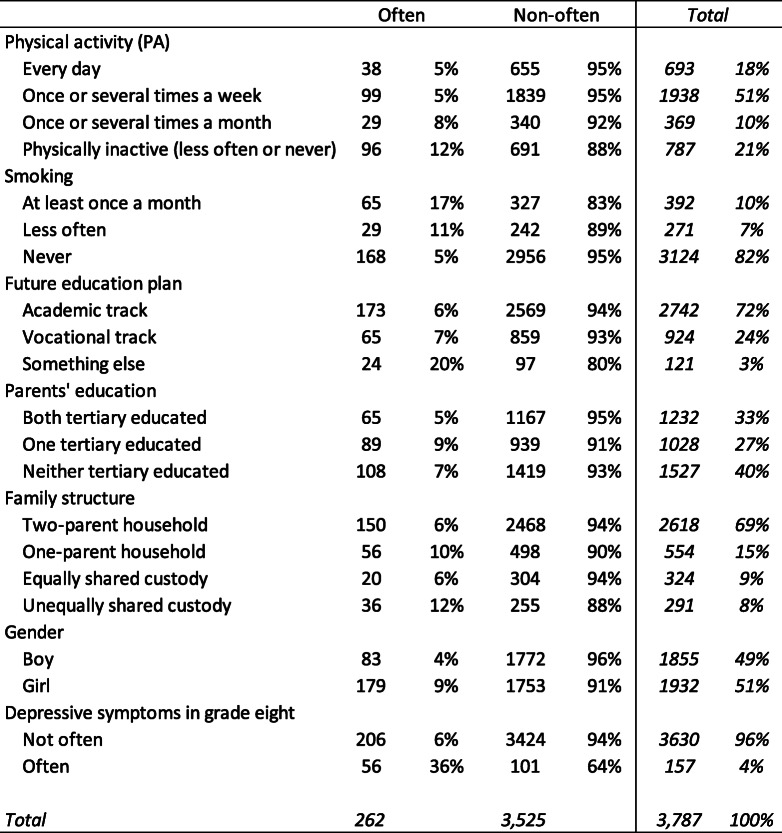
Descriptive statistics of variables used for logistic regression model

*Source: Authors’ calculations based on CILS4EU Sweden*

Table 2 shows that 18% of our respondents participated in PA every day, 51% participated in PA once or several times a week, 10% participated in PA once or several times a month, and 21% were physically inactive. Among those who participated in PA on a daily or weekly base, 5% often felt depressed. Among those who participated in PA on a monthly base, 8% often felt depressed. Among the physically inactive group, 12% often felt depressed. These descriptive statistics suggest a connection between physical inactivity during leisure time and higher levels of depressive symptom.

Among respondents who never smoked, 5% often felt depressed. Among those who smoked at least once a month, 17% often felt depressed. Among those who planned to go for upper secondary education (independent of education track), 6–7% often felt depressed. Among those who planned for something else, though amounting to only 3% of our total sample, 20% often felt depressed. Among adolescents with one parent tertiary educated, 9% often felt depressed, which was slightly higher than among the other groups (5 and 7%, respectively). Adolescents living in one-parent households or under unequally shared custody had the highest proportions (10 and 12%, respectively) of often feeling depressed.

Our sample was evenly distributed across gender, with 49% boys and 51% girls. Four percent of boys and 9% of girls often felt depressed. Among adolescents who often felt depressed during grade eight, 36% showed depressive symptoms often after they transited to grade nine.

### Estimated results: association between leisure time physical activity and symptoms of depression

Table 3 displays the estimated odds ratios of often feeling depressed from the logistic regression model. Model 1, the simplest model, includes only physical activity to capture the unconditional association between participation in PA during leisure time and depression. From Models 2 to 3, smoking and other factors were added in a stepwise manner. In Model 4, we controlled for depressive symptoms at baseline in the analysis.

**Table 3:**
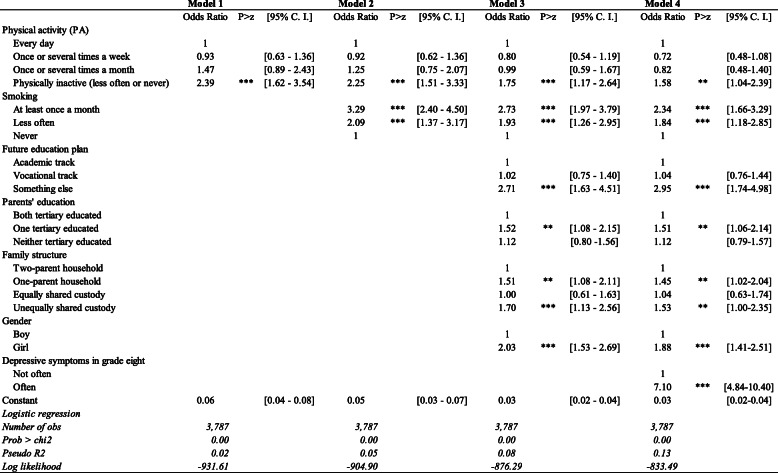
Binary logistic regression. Odds ratios of often feeling depressed among adolescents in Sweden, 2012

Note: Statistical significance: ***p < .01; ** .01 < p < .05; and * .05 < p < .10

*Source*: *Authors’ calculations based on CILS4EU Sweden*

Model 1 shows that relative to adolescents who participated in PA every day, those who participated in PA once or several times a week or a month did not show a significant difference in the likelihood of often feeling depressed. Notably, relative to the same group, physically inactive adolescents had 139% higher odds of often feeling depressed.

When smoking and other social-demographic factors were added in Models 2–3, the odds of reporting symptoms of depression for the physically inactive group remained significantly higher. All else being equal, relative to adolescents who participated in PA every day, those who were physically inactive had 75% higher odds of often feeling depressed. When adolescents’ depressive symptoms at baseline was involved into the model (see Model 4), those who were physically inactive had 58% higher odds of often feeling depressed than the daily exercisers. These findings suggest that regular participation in PA on a daily, weekly, or monthly basis was associated with better mental health. Physical inactivity was associated with significantly higher odds of poor mental health.

Our estimates for other variables also showed noteworthy results. Model 4 showed that relative to the non-smokers, those who smoked at least once a month had 134% higher odds of often feeling depressed; those who smoked less often had 84% higher odds of often feeling depressed.

The odds of often feeling depressed among adolescents who planned to follow the vocational track in upper secondary school did not differ from that of those who planned to pursue an academic track. Nonetheless, compared to adolescents who planned to go for academic or vocational upper secondary education, those without such plan - mainly those who wanted to work or do something else - had approximately 195% higher odds of often feeling depressed. The adolescents without a future education plan may be a selected group (amounting to only 3% of the total sample). Lacking plans for the future, they might be more vulnerable to poor mental health.

Whether parents were both highly educated or both low educated did not make a difference. However, adolescents with one parent tertiary educated while the other not had notably higher odds of often feeling depressed. Compared to educationally homogenous couples, educationally heterogeneous couples may be more likely to confront communication problems or to have value conflicts. Accordingly, children of educationally heterogeneous couples may have higher chance of being exposed to parental conflict, which plays a negative role in children’s mental health development.

As regards family structure the results show that living with both parents in the same household or living under equally shared custody of parents in two different households were associated with fewer symptoms of depression among adolescents. Living with only one parent or living under unequally shared custody significantly increased adolescents’ likelihood of often feeling depressed. These findings signal that having equal access to both parents may be beneficial for adolescents’ mental health, whereas having less or no contact with one parent is associated with poorer mental health.

Girls were more vulnerable than boys in terms of internalizing mental health problems. Relative to boys, girls’ odds of often feeling depressed was about 88% higher.

Lastly, compared to adolescents who did not often feel depressed during grade eight, those who often felt depressed during grade eight had six times higher odds of often feeling depressed after they transited to grade nine.

To investigate how the association between leisure time PA and depressive symptoms varied across genders, we estimated the interactive effect of physical activity frequency and gender, while other variables were controlled for. Our likelihood ratio test did not show any significant improvement of the model fit, indicating that there was no significant interactive effect of physical activity and gender.

In order to check the robustness of our results, we executed a number of parallel analyses. In one, we controlled for adolescents’ physical activity in grade eight in our analysis. The estimation did not show any significant results. In addition, given that our sample is over-represented by adolescents with foreign-born parents, we also controlled for parents’ migration background in the analysis. The estimated results for adolescents with foreign-born parents did not differ significantly from those of adolescents with native-born parents. Moreover, controlling for parents’ migration background did not significantly improve the model fit.

## Discussion

This study explored how leisure time PA frequency was associated with depressive symptoms among adolescents in Sweden. Our results show that adolescents who participated in PA every day, once or several times a week, and once or several times a month had a substantially lower odds of often feeling depressed than those who were physically inactive during leisure time. The odds of experiencing symptoms of depression among those who participated in PA on a weekly or monthly basis did not differ significantly from those who were physically active on a daily basis. This pattern applied to both boys and girls. Further, girls were more likely than boys to often feel depressed.

Although we did not use identical measures for PA and depressive symptoms, our results in the Swedish context resemble those in Finland and Iceland, where physical inactivity was strongly associated with mental health problems among both boys and girls. Our results differ from those in Denmark, where participation in PA was only associated with mental health among girls. In addition, our results are different from those in the Norwegian context where the association between physical activity and mental health was weak and only a high amount of physical activity was positively related to mental health. In Sweden, even monthly participation in PA (at least once a month) was positively associated with adolescents’ mental health.

### Implications

The role of physical activity in improving mental health has recently drawn increasing attention in many high-income countries [16]. Our findings in the Swedish context suggest that participation in PA on a daily, weekly and monthly basis is a positive behavior that can yield psychological benefits among adolescents. Those who were physically inactive during leisure time, namely those who participated in PA less than monthly or never, may be at risk for experiencing symptoms of depression. From a public health perspective, a knowledge of the form, type and motives of physical activity is likely to be important in understanding mental health outcomes in adolescents [16]. This study contributes to existing knowledge by bringing forth frequency of physical activity. We hereby appeal for social actions to engage more adolescents in physical activity during leisure time. This may be particularly relevant for girls.

### Limitations

The understanding of our findings warrants awareness of the following issues. The outcome measure of the study was based on only one question about depressive symptoms. Boys may have a tendency to underreport their emotional problems [25], which may have biased our results.

Further, this study addressed the association between participation in PA and symptoms of depression - one common internalizing mental health problems. The findings cannot be generalized to the association between physical activity and other mental health problems, such as externalizing problems like antisocial behavior.

In addition, though CILS4EU is a longitudinal survey, this study is cross-sectional as the key variables used in our analysis such as physical activity and depression were drawn only from the second survey wave. Hence, our results can only denote an association between physical activity and depression rather than any causal inference. It is possible that regular participation in physical activity may decrease the level of depressive symptoms. Inversely, experiencing symptoms of depression may negatively influence adolescents’ frequency of physical activity.

Finally, there may be other factors of interest that we were not able to include in our analyses. For example, boys may show more active interest in physical activity than girls, which may have influenced our results. Further, types of physical activity, such as team or individual sports, as well as childhood experiences may also play a role [16, 17]. Unfortunately, we were not able to address these issues due to limitations of our data. Future research may consider these issues if relevant data become available.

## Conclusions

Physical wellness and mental fitness go hand in hand. The findings of this study suggest that regular participation in physical activity during leisure time is a positive behavior associated with psychological benefits among adolescents, independent of gender. This study hereby calls for recognition of the value of physical activity in promoting adolescents’ mental health.

## Data Availability

The dataset supporting the conclusions of this article - CILS4EU - is available from GESIS Data Archive for the Social Sciences, Cologne, Germany (http://www.cils4.eu/). The data are available to the international research community for public use.

## Abbreviations

CI: Confidence interval
PA: Physical activity
